# Severe HELLP syndrome masquerading as thrombocytopenic thrombotic purpura: a case report

**DOI:** 10.1186/s12882-020-01865-y

**Published:** 2020-05-29

**Authors:** Cyril Mousseaux, Bérangère S. Joly, Inna Mohamadou, Romain Arrestier, Alexandre Hertig, Cédric Rafat

**Affiliations:** 1 Department of Nephrology, CHU Tenon, Assistance Publique-Hopitaux de Paris, Paris, France; 2 Hematology Laboratory, French Reference Center for Thrombotic Microangiopathies, Hôpital Lariboisière, Assistance Publique-Hôpitaux de Paris, Paris, France

**Keywords:** Acute liver failure, HELLP syndrome, Therapeutic plasma exchange, Thrombotic microangiopathy, Thrombotic thrombocytopenic purpura, Case report

## Abstract

**Background:**

Thrombotic microangiopathies (TMAs) occurring in the postpartum period may be difficult to manage. They present as the combination of mechanical hemolytic anemia and consumption thrombocytopenia due to endothelial dysfunction. The cause of this endothelial aggression can be multiple: thrombocytopenic thrombotic purpura (TTP), HELLP syndrome, antiphospholipid syndrome, atypical hemolytic and uremic syndrome or acute fatty liver of pregnancy. TTP results from a severe deficiency of ADAMTS13, which is a protease cleaving specifically von Willebrand factor chiefly produced by liver cells. There are two main causes, the production of anti-ADAMTS13 auto-antibodies and, more rarely, a genetic deficiency in ADAMTS13. First-line treatment is based on plasma exchange. HELLP syndrome occurs in the third trimester of pregnancy usually in association with preeclampsia and represents a form of TMA characterized by damage to the sinusoidal capillaries of the liver. Prompt delivery is the main treatment. We present a case illustrating the challenges in discriminating between different postpartum TMAs, with a focus on the distinction between TTP and HELLP syndrome. Specifically, we highlight how acute liver failure (ALF) stemming from HELLP may lead to TTP with a spectacular response to plasma exchanges.

**Case:**

A 28-year-old, 33 + 4 weeks pregnant woman presented with severe preeclampsia complicated by ALF in the setting of partial liver necrosis, disseminated intravascular coagulation, microangiopathic hemolytic anemia and acute kidney injury. Greatly diminished levels of ADAMTS13 (< 5%) activity and neurological impairment suggested an initial diagnosis of thrombotic thrombocytopenic purpura (TTP). Therapeutic plasma exchange (TPE) was initiated and complete renal, neurological, hematological and hepatic recovery was observed. Secondary TTP induced by ALF due to HELLP syndrome was the final diagnosis.

**Conclusion:**

Our case addresses the overlapping nature of postpartum TMAs and raises the possibility that HELLP-induced ALF may constitute an additional mechanism resulting in TTP, thereby opening a possible indication for TPE.

## Background

Postpartum thrombotic microangiopathy (TMA) clusters multiple clinical entities with largely overlapping clinical presentation albeit with distinct physiopathology and requiring specific management. They include thrombocytopenic purpura (TTP), HELLP syndrome, antiphospholipid syndrome, atypical hemolytic and uremic syndrome or acute fatty liver of pregnancy. TTP is a rare and life-threatening TMA characterized by a severe deficit in ADAMTS13 (disintegrin and metalloprotease with thrombospondin type 1 repeats, member 13) [1] either of anti-ADAMTS13 autoimmune etiology or genetic mutation of ADAMTS13. During pregnancy, TTP occurs primarily during the second and third semesters and genetic forms are more prevalent. Therapeutic plasma exchange (TPE) and corticosteroids form the mainstay of first line therapeutic management which may be complemented by rituximab and caplacizumab in refractory cases [2]. In case of concurrent preeclampsia, the favored diagnosis is HELLP syndrome which consists of peripheral signs of TMA associated with liver involvement (mainly liver cytolysis) and renal injury. In most situations, delivery and a supportive treatment prove to be curative. Exceptionally, HELLP syndrome may result in liver necrosis, and *in fine* acute liver failure (ALF). Herein, we present the case of a preeclamptic patient who developed liver necrosis following HELLP syndrome that was practically indistinguishable from TTP but which was successfully treated with TPE.

## Case presentation

A 28-year-old woman was admitted for severe preeclampsia at 33 + 4 weeks. It was her fifth pregnancy. None of her previous pregnancies had been complicated by preeclampsia. Her foetus presented with severe intrauterine growth restriction (estimated foetal weight < 3rd percentile).

Her initial physical examination was remarkable for hypertension and a dipstick proteinuria (2+). She had no neurological symptoms.

Initial laboratory investigations (Table 1) revealed mild renal impairment, hyperleukocytosis, mild anemia without thrombocytopenia. Coagulation assays were unremarkable.

**Table 1:** Laboratory investigations prior to Cesarean section to the intensive care unit (ICU) and to our unit

Variable	Normal range	Prior to Cesarean	Admission to the ICU	Admission to our unit
**Hematology and coagulation assays**				
***Hemoglobin (g/dL)***	**12–16**	**11.7**	**12.6**	**6**
*White cell count (per μL)*	4500–10,000	21,000	27,600	30,000
*Differential count (%)*				
*Neutrophils*	40–70	76	76.7	83
*Others*	30–70		24.3	17
***Platelet count (per μL)***	**150,000–400,000**	**160,000**	**155,000**	**53,000**
***Fibrinogen (g/liter)***	**2–6**	**2.41**	**2**	**4.59**
*Schizocytes (%)*	< 1	0	Not specified	**2**
*Prothrombin time (%)*	70–110	85	61	58
***Factor V (%)***	**70–120**	**75**	**24**	**70**
*Activated partial thromboplastin time ratio*	0.8–1.2	1.2	1.21	1.31
*D-dimers*	< 500		> 20,000	
**Renal analysis**				
***Creatinine (mg/dL)***	**0.6–1.5**	**1.1**	**1.56**	**3.7**
*Blood urea nitrogen (mg/dL)*	8–25		14	38.8
**Hepatic analysis**				
*Albumin (g/L)*	35–50		25	21
***Lactate dehydrogenase (U/L)***	**125–250**		**5000**	**7820**
***Haptoglobin (g/L)***	**0.2–0.4**		**0.2**	**< 0.08**
*Aspartate aminotransferase (U/L)*	< 35		Hemolysis	1749
*Alanine aminotransferase (U/L)*	< 45		500	833
*Alkaline phosphatase (U/L)*	< 115		229	283
*Gamma glutamyl transpeptidase (U/L)*	< 45		168	212
*Bilirubin total/conjugate (mg/dL)*	0.3–1.9 / 0–0.3		13.7 / 8.9	40 / 28.2
*Arterial lactate (mmol/L)*	0–2		1.9	2.2
**TMA exploration**				
***ADAMTS13 activity (%)***	**50–150**			**< 5%**
***IgG anti-ADAMTS13***				**Negative**
*Coombs test*	Negative		Negative	
*HIV test*			Negative	
*C3 (g/L)*	0.9–1.8		0.56	
*C4 (g/L)*	0.1–0.4		0.06	
*Antinuclear and anti-DNA antibodies*			Negative	
*Factor H (%)*	65–140		106	
*Factor I (%)*	70–130		125	
*Factor H autoantibody*			Negative	
*Antiphospholipid antibodies*^a^			Negative	

^a^Antiphospholid antibodies: anticardiolipin, beta-2 glycoprotein I (β2GPI), and lupus anticoagulant

Two days after admission, at 33 + 6 weeks, she presented with epigastric pain and her foetus was bradycardic (80 beats per minute). An emergency Cesarean section was performed during which a large retroplacental hematoma was uncovered. She delivered a 1400 g healthy baby boy. Postpartum hemorrhage (1.2 L) occurred immediately prompting the administration of 3 L of crystalloid fluids, and 1 g of tranexamic acid. In order to prevent eclampsia, treatment with magnesium sulfate was initiated.

Due to severe preeclampsia and anuria following the Cesarean section, she was transferred to the intensive care unit (ICU) for monitoring.

Physical examination revealed no fever (37.7 °C), controlled blood pressure (138/84 mmHg under 4 g per hour of intravenous nicardipine) and no tachycardia (97 beats per minute). Neurologic examination was remarkable for drowsiness (Glasgow score = 13) with no evidence of focal neurological signs and normal osteotendinous reflexes. Epigastric pain was persistent.

Immediately after surgery, hemostasis disorders consistent with disseminated intravascular coagulation (DIC) appeared (Table 1) with an International Society on Thrombosis and Hemostasis score of seven, prompting the administration of five units of red blood cells, three units of fresh frozen plasma, and 3 g of fibrinogen. At this point, prothrombin time fell to 46%, platelets and fibrinogen decreased to 23.000 and 1.92 g/L respectively, and D-dimer value was above 20.000 μg/L.

Signs of HELLP syndrome were present*,* as defined by severe cytolysis, thrombocytopenia and microangiopathic hemolytic anemia (Table 1). ALF developed, as defined by grade 3 encephalopathy, and a MELD score of 32. Blood glucose test remained normal, but a peak lactate level of 7 mmol/L was observed on day 2. Simultaneously, an acute kidney injury (KDIGO stage 3) occurred (Table 1) requiring hemodialysis (overload and anuria). Finally, empiric antibiotic therapy with tazocillin was started due to suspected sepsis with fever plateau at 38.5.

**Fig. 1:**
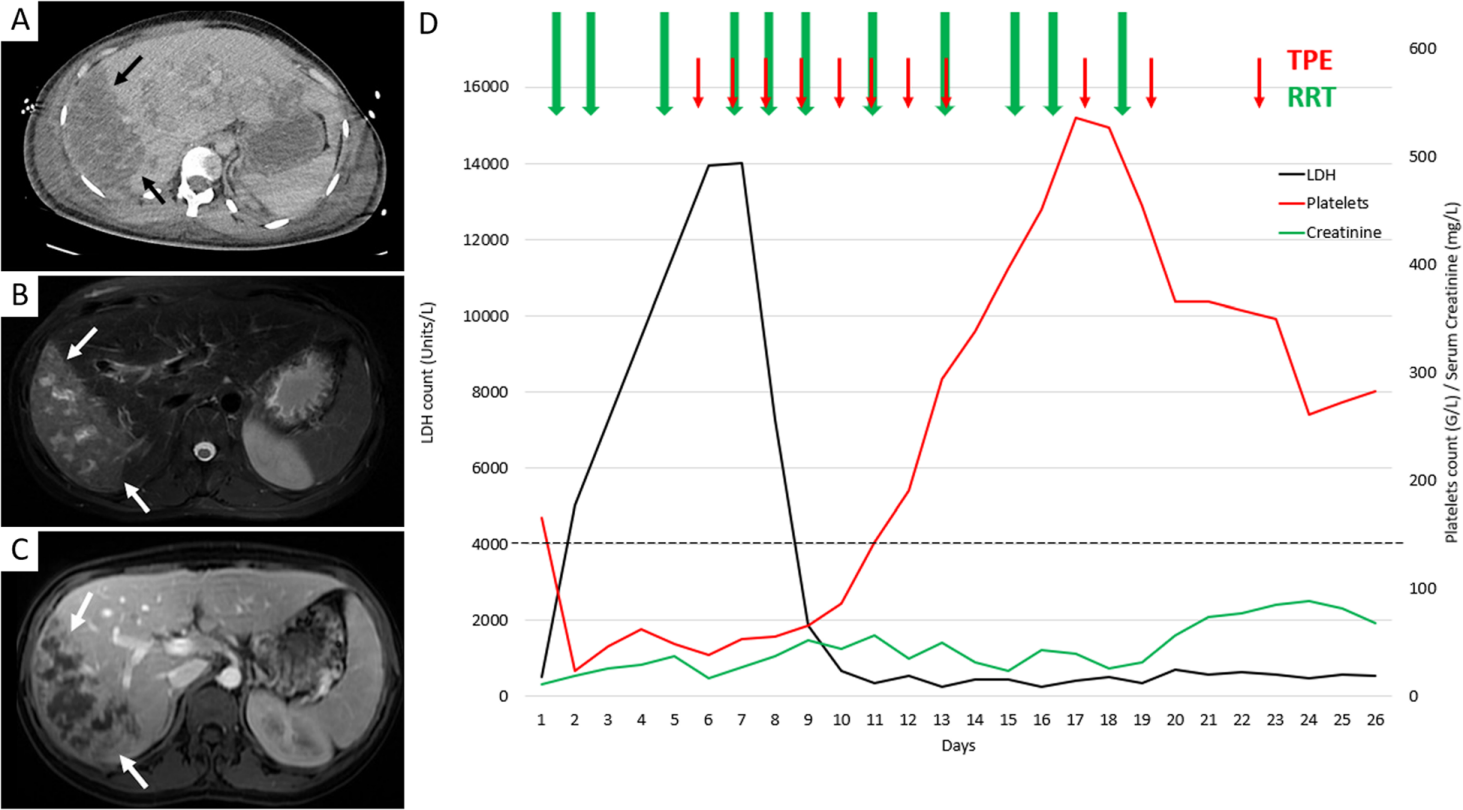
Imaging and temporal trends of lactate dehydrogenase (LDH) levels and platelets following delivery*.* Computerized Tomography scan (**a**) with axial reconstruction shows lack of perfusion of the posterior sector of the liver (black arrow). Magnetic Resonance Imaging with T2 sequence (**b**) and T1 sequence with gadolinium injection (**c**) show high signal T2 of the posterior sector of the liver related to necrosis (white arrows) and lack of enhancement after gadolinium injection (white arrows) related to the infarction of the hepatic parenchyma. Therapeutic plasma exchange (TPE, red arrows) was initiated at day 5 following delivery and renal replacement therapy (RRT, green arrows) at day 1. Serum lactate dehydrogenase (LDH, black line), platelet count (red line) and serum creatinine (green line, mg/L) are given on the ordinate. Day 1 is the day of delivery. The dotted line is the standard for platelets

Four days following delivery, the patient was referred to our renal ICU due to suspected TTP. The neurological examination was unchanged with persistent drowsiness. A brain magnetic resonance imaging (MRI) was deemed normal. Abdominal MRI showed heterogeneous high signal intensity on T1 and T2-weighted images in the posterior right lobe of the liver suggestive of liver necrosis (Fig. 1a, b, c).

In the hypothesis of a coincident TTP, methylprednisolone (1 mg/kg) and daily TPE with 100% frozen plasma units were initiated. By the fourth TPE, liver function and platelet count were restored to normal values (Fig. 1d). TPE was thereafter gradually tapered off with a total of 11 sessions (8 daily sessions, then two sessions spaced 48 h apart and one last session 4 days later).

As part of the diagnostic work-up, ADAMTS13 activity was found to be undetectable, yet anti-ADAMTS13 auto-antibodies were not found. Corticosteroids were stopped as soon as ADAMTS13 auto-antibody negativity was obtained (total duration of 9 days). A kidney biopsy demonstrated severe acute tubular necrosis but no signs of cortical necrosis or TMA. Atypical hemolytic and uremic syndrome was excluded from renal biopsy and alternate complement pathway explorations, including genetic analysis (Table 1). Acute fatty liver of pregnancy was ruled out given the presence of hemolysis, and the absence of hypoglycemia and hyperechogenicity on liver ultrasound. Antiphospholipid antibody syndrome was also ruled out.

ADAMTS13 activity rose to 17 and 24%, 4 and 7 days, respectively, after discontinuation of TPE. Genetic analysis of ADAMTS13 did not reveal any sequence variation. Both antibody-mediated TTP and congenital TTP were thus excluded. On hospital discharge (27 days after delivery) the patient had recovered complete liver and kidney function.

## Discussion and conclusion

Our case demonstrates that ALF caused by a HELLP syndrome may be severe enough to induce severe ADAMTS13 deficiency and elicit the full range of clinical symptoms associated with TTP. Akin to the autoimmune and congenital forms of the condition, this cause of secondary TTP is also amenable to TPE. Yet its timely recognition and differentiation from the canonical forms of TTP is essential to avoid unnecessary and potentially harmful treatments such as immunosuppressive therapy, a mainstay in the auto-immune form of the disease, or repeated prophylactic TPE, as considered in some cases of congenital TTP.

The typical course of platelets during a HELLP syndrome has been previously described [3]. Here, the persistence of TMA features, along with neurological impairment, encouraged us to prioritize a diagnosis of TTP and to initiate TPE.

Central to these investigations is the assessment of ADAMTS13 activity since severe functional deficiency in ADAMTS13 (< 10%) serves as the indisputable pathophysiological basis for TTP. Reciprocally, severe ADAMTS13 deficiency is held to be the only specific, if not quasi-pathognomonic, diagnostic marker of the disease [1]. TTP may be caused by either IgG auto-antibodies directed against ADAMTS13 or by a recessively inherited bi-allelic mutation of the *ADAMTS13* gene. In adulthood, this latter mechanism is more likely to occur during pregnancy than in other settings [4].

This case contends that a third mechanism may be involved: the severity of liver failure, as evidenced by the lowered levels of factor V (30%) along with partial liver necrosis. Because of the dual blood supply of the liver, liver necrosis is an exceptional complication of HELLP syndrome [5]. A retrospective analysis derived from the UNOS databank yielded only eight cases of fulminant liver failure from HELLP syndrome requiring liver transplantation [6].

Therefore, the diagnosis hinged on whether the patient presented with genuine TTP, or whether HELLP syndrome may have precipitated the collapse in ADAMTS13 levels. The fact that the flare of TMA was observed postpartum (in contrast with what is typically observed in patients with primary TTP) [7], and that the context was of a preeclampsia-related liver injury, argued for such a scenario. Moreover, TTP is not usually associated with fetal abnormality because placenta function is preserved.

In patients with HELLP syndrome, low levels of ADAMTS13 have indeed been reported but, to our knowledge, never in the critical range (< 10%) where TTP should be regarded as a superimposed diagnosis. In fact, in one series which specifically focused on ADAMTS13 assessment in patients with HELLP syndrome, the lowest recorded ADAMTS13 level was 12% [8]. In two other case series, two patients diagnosed initially with HELLP syndrome did exhibit ADAMTS13 levels below 5%, but in both cases the final diagnosis was later revised to congenital TTP [9, 10].

Liver function integrity is a prerequisite for normal ADAMTS13 production [11]. Accordingly, ADAMTS13 plasma levels have been shown to be inversely correlated to the severity of liver injury both in the chronic setting of liver cirrhosis and in ALF [12]. In various conditions other than HELLP syndrome, ALF has been shown to be associated with greatly diminished ADAMTS13 levels, below the threshold required for TTP diagnosis.

Another prominent feature in this case was the occurrence of severe DIC. DIC has been proposed as a possible cause of ADAMTS13 [13] consumption although some authors have suggested that sepsis-induced inflammation may in fact be the key trigger in reducing the levels of ADAMTS13 [14].

Rather than HELLP syndrome per se*,* it is therefore plausible that the direct and indirect clinical complications of HELLP syndrome, most importantly ALF, with the possible contribution of DIC and sepsis, may have coincided to reduce ADAMTS13 levels to such an extent that TTP was genuinely manifested, especially against the backdrop of neurological impairment. However, the absence of antibodies against ADAMTS13, as well as the rebound of ADAMTS13 levels following recovery and the absence of genetic abnormalities in the *ADAMTS13* gene prompted us to retain the final diagnosis of secondary TTP resulting from HELLP-induced ALF. Given the initial hypothesis of severe TTP, TPE was initiated. Within 24 h there was such an extensive improvement in the patient’s neurological status as well as in LDH and platelet levels (Fig. 1d) that these were originally interpreted as further evidence supporting this diagnosis. TPE has previously been investigated in the context of HELLP syndrome with several studies reporting accelerated recovery following TPE initiation [15]. However, most studies were observational and conducted at a time when ADAMTS13 assessment was not readily available, thus suggesting a possible case mix effect. Likewise, the indication of TPE has also been examined in ALF with encouraging results [16]. Finally, although TPE was originally believed to be possibly detrimental in the setting of sepsis, it has been shown recently that such patients may in fact safely benefit from TPE, and even more so when DIC is present [17].

Although seemingly disparate, TPE plays a similar role in these conditions, namely to replenish the levels of ADAMTS13, to correct coagulopathy, provided that fresh frozen plasma is used, and perhaps to mitigate the inflammatory cascade. Remarkably, the patient encapsulated all these features.

Taken together, this case should inspire clinicians to move away from the common heuristic whereby a satisfactory response to TPE provides indisputable evidence in favor of auto-immune or congenital TTP. More importantly, it begs the question as to whether plasma exchange may benefit a subset of patients with severe HELLP syndrome with markedly diminished levels of ADAMTS13.

## Data Availability

If required, the relevant material can be provided by corresponding author on reasonable request.

## Abbreviations

ADAMTS13: Disintegrin and metalloprotease with thrombospondin type 1 repeats, member 13
ALF: Acute liver failure
DIC: Disseminated intravascular coagulation
MRI: Magnetic resonance imaging
TMA: Thrombotic microangiopathies
TPE: Therapeutic plasma exchange
TTP: Thrombocytopenic thrombotic purpura
